# Acid–base status and its clinical implications in critically ill patients with cirrhosis, acute-on-chronic liver failure and without liver disease

**DOI:** 10.1186/s13613-018-0391-9

**Published:** 2018-04-19

**Authors:** Andreas Drolz, Thomas Horvatits, Kevin Roedl, Karoline Rutter, Richard Brunner, Christian Zauner, Peter Schellongowski, Gottfried Heinz, Georg-Christian Funk, Michael Trauner, Bruno Schneeweiss, Valentin Fuhrmann

**Affiliations:** 1 Division of Gastroenterology and Hepatology, Department of Internal Medicine III, Medical University of Vienna, Vienna, Austria; 2 Department of Intensive Care Medicine, University Medical Center, Hamburg-Eppendorf, Martinistraße 52, 20246 Hamburg, Germany; 3 Division of Oncology and Infectious Diseases, Department of Internal Medicine I, Medical University of Vienna, Vienna, Austria; 4 Division of Cardiology, Department of Internal Medicine II, Medical University of Vienna, Vienna, Austria; 5 Department of Respiratory and Critical Care Medicine, and Ludwig Boltzmann Institute for COPD, Otto-Wagner Hospital, Vienna, Austria

**Keywords:** Acid–base, Cirrhosis, Acute-on-chronic liver failure, Mortality

## Abstract

**Background:**

Acid–base disturbances are frequently observed in critically ill patients at the intensive care unit. To our knowledge, the acid–base profile of patients with acute-on-chronic liver failure (ACLF) has not been evaluated and compared to critically ill patients without acute or chronic liver disease.

**Results:**

One hundred and seventy-eight critically ill patients with liver cirrhosis were compared to 178 matched controls in this post hoc analysis of prospectively collected data. Patients with and without liver cirrhosis showed hyperchloremic acidosis and coexisting hypoalbuminemic alkalosis. Cirrhotic patients, especially those with ACLF, showed a marked net metabolic acidosis owing to increased lactate and unmeasured anions. This metabolic acidosis was partly antagonized by associated respiratory alkalosis, yet with progression to ACLF resulted in acidemia, which was present in 62% of patients with ACLF grade III compared to 19% in cirrhosis patients without ACLF. Acidemia and metabolic acidosis were associated with 28-day mortality in cirrhosis. Patients with pH values < 7.1 showed a 100% mortality rate. Acidosis attributable to lactate and unmeasured anions was independently associated with mortality in liver cirrhosis.

**Conclusions:**

Cirrhosis and especially ACLF are associated with metabolic acidosis and acidemia owing to lactate and unmeasured anions. Acidosis and acidemia, respectively, are associated with increased 28-day mortality in liver cirrhosis. Lactate and unmeasured anions are main contributors to metabolic imbalance in cirrhosis and ACLF.

**Electronic supplementary material:**

The online version of this article (10.1186/s13613-018-0391-9) contains supplementary material, which is available to authorized users.

## Background

Derangements in acid–base balance are frequently observed in critically ill patients at the intensive care unit (ICU) and present in various patterns [1–4]. Severe acid–base disorders, especially metabolic acidosis, have been associated with increased mortality [5, 6]. As a consequence, acid–base status in critically ill patients with various disease entities has been extensively studied.

Yet, only a few studies assessed the impact of underlying chronic liver disease on acid–base equilibrium in critical illness [7, 8]. While a balance of offsetting acidifying and alkalinizing metabolic acid–base disorders with a resulting equilibrated acid–base status has been described in stable cirrhosis [9], severe derangements with resulting net acidosis owing to hyperchloremic, dilutional and lactic acidosis were observed when cirrhosis was accompanied by critical illness [7, 8]. Acute liver failure (ALF) is characterized by a different acid–base pattern with dramatically increased lactate levels [10]. The acidifying effect of this increase in lactate was neutralized by hypoalbuminemia in non-paracetamol-induced ALF [11].

Despite advantages in intensive care medicine, which have led to an improved outcome over the last decade [12], mortality in cirrhotic patients admitted to ICU is still high [13–15]. Measurement and knowledge of specific acid–base patterns and their implications in critically ill patients with liver cirrhosis may help to improve patient management, especially in the ICU setting [16]. However, to our knowledge, the acid–base profile of critically ill cirrhotic patients with acute-on-chronic liver disease (ACLF) has not been compared to critically ill patients without acute or chronic liver disease. Most information on the acid–base status of critically ill patients with cirrhosis was obtained by comparing these patients with healthy controls [8]. Yet, part of metabolic disturbances in critically ill patients with liver cirrhosis may be attributable to critical illness per se, rather than to the presence of chronic liver disease.

The aim of this study was to assess acid–base patterns of critically ill patients with liver cirrhosis and ACLF, respectively, in comparison with critically ill patients without acute or chronic liver disease.

## Methods

### Patients

All patients admitted to 3 medical ICUs at the Medical University of Vienna between July 2012 and August 2014 were screened for inclusion in the study. For the present study, only patients who had arterial blood samples drawn within 4 h after ICU admission were eligible for inclusion. Patients with acute liver injury in the absence of chronic liver disease were excluded. One hundred and seventy-eight patients with liver cirrhosis were identified as eligible for inclusion. The control group of 178 critically ill patients without acute or chronic liver disease was selected by propensity score matching (PSM).

On admission, Simplified Acute Physiology Score II (SAPS II) [17], SOFA [18], infections and organ dysfunctions were documented.

All patients were screened for the presence of acute kidney injury (AKI) defined by urine output and serum creatinine according to the Kidney Disease: Improving Global Outcomes (KDIGO) Clinical Practice Guidelines for Acute Kidney Injury [19].

The presence of liver cirrhosis was defined by a combination of characteristic clinical (ascites, caput medusae, spider angiomata, etc.), laboratory and radiological findings (typical morphological changes of the liver, sings of portal hypertension, etc., in ultrasonography or computed tomography scanning), or via histology, if available. ACLF was identified and graded according to recommendations of the chronic liver failure (CLIF) consortium of the European Association for the Study of the Liver (EASL) [20]. CLIF-SOFA score [20] and CLIF-C ACLF score [21] were calculated. Septic shock was defined according to the recommendations of the Surviving Sepsis Campaign [22].

Twenty-eight-day mortality and 1-year mortality were assessed on site or by contacting the patient or the attending physician, respectively.

This study is based on a post hoc analysis of prospectively collected data [23]. The Ethics Committee of the Medical University of Vienna waived the need for informed consent due to the observational character of this study.

### Sampling and blood analysis

On admission, arterial blood samples were collected from arterial or femoral artery and parameters for the assessment of acid–base status were instantly measured.

pH, partial pressure of carbon dioxide (PaCO_2_), ionized calcium (Ca^2+^) and lactate were measured with a blood gas analyzer (ABL 725; Radiometer, Copenhagen, Denmark). Samples of separated plasma were analyzed for concentrations of sodium (Na^+^), potassium (K^+^), chloride (Cl^−^), magnesium (Mg^2+^), inorganic phosphate (Pi), albumin (Alb), plasma creatinine, blood urea nitrogen (BUN), aspartate aminotransferase (AST) and alanine aminotransferase (ALT) by a fully automated analyzer (Hitachi 917; Roche Diagnostics GmbH, Mannheim, Germany). Na^+^ and Cl^−^ were measured using ion-selective electrodes. Lactate was measured with an amperometric electrode.

### Acid–base analysis

Arterial concentration of bicarbonate (HCO3^−^) was calculated from measured pH and PaCO_2_ values according to the Henderson–Hasselbalch equation [24, 25]. Base excess (BE) was calculated according to the formulae by Siggaard-Andersen [24–26].

Quantitative physical–chemical analysis was performed using Stewart’s biophysical methods [27], modified by Figge and colleagues [28].

Apparent strong ion difference (SIDa) was calculated:

$$\begin{aligned} {\text{SIDa}} &= {\text{Na}}^{ + } + {\text{K}}^{ + } + 2 \times {\text{Mg}}^{2 + } + 2 \times {\text{Ca}}^{2 + } - {\text{Cl}}^{ - } - {\text{lactate}} \\ & \left( {{\text{SIDa}}\;{\text{in}}\;{\text{mEq/l;}}\;{\text{all}}\;{\text{concentrations}}\;{\text{in}}\;{\text{mmol/l}}} \right) \\ \end{aligned}$$

Effective strong ion difference (SIDe) was calculated in order to account for the role of weak acids [29]:

$$\begin{aligned} {\text{SIDe}} & = 1000 \times 2.46 \times 10^{ - 11} \times \frac{{{\text{PaCO}}_{2} }}{{10^{{ - {\text{pH}}}} }} + {\text{Alb}} \times \left( {0.123 \times {\text{pH}} - 0.631} \right) + {\text{Pi}} \times \left( {0.309 \times {\text{pH}} - 0.469} \right) \\ & \left( {{\text{SIDe}}\;{\text{in}}\;{\text{mEq/l;}}\;{\text{PaCO}}_{ 2} \;{\text{in}}\;{\text{mmHg,}}\;{\text{Alb}}\;{\text{in}}\;{\text{g/l}}\;{\text{and}}\;{\text{Pi}}\;{\text{in}}\;{\text{mmol/l}}} \right) \\ \end{aligned}$$

The effect of unmeasured charges was quantified by the strong ion gap (SIG) [30]:

$$\begin{aligned} {\text{SIG}} = {\text{SIDa}} - {\text{SIDe}} \hfill \\ \left( {{\text{all}}\;{\text{parameters}}\;{\text{in}}\;{\text{mEq/l}}} \right) \hfill \\ \end{aligned}$$

Based on the concept that BE can be altered by plasma dilution/concentration reflected by sodium concentration (BE_Na_), changes of chloride (BE_Cl_), albumin (BE_Alb_), lactate (BE_Lac_) and unmeasured anions (BE_UMA_), the respective components contributing to BE were calculated according to Gilfix et al. [31]. The detailed formulae for the BE subcomponents are shown in “Appendix.”

Thus, total BE is calculated by the sum of the BE subcomponents:

$${\text{BE}} = {\text{BE}}_{\text{Na}} + {\text{BE}}_{\text{Cl}} + {\text{BE}}_{\text{Alb}} + {\text{BE}}_{\text{Lac}} + {\text{BE}}_{\text{UMA}}$$

Reference values were obtained from a historical cohort of healthy volunteers, as published elsewhere [8]. Acidemia and alkalemia were defined by pH < 7.36 and > 7.44, respectively. HCO3^−^< 22 and > 26 mmol/l, respectively, defined metabolic acidosis and alkalosis [2]. Respiratory acidosis and alkalosis were identified by PaCO_2_ > 45 and < 35 mmHg, respectively. BE_Na_ < − 5 and > 5 mmol/l defined dilutional acidosis and alkalosis, respectively. Hyperchloremic acidosis and hypochloremic alkalosis were defined by BE_Cl_ < − 5 and > 5 mmol/l, respectively. BE_Alb_ > 5 mmol/l identified hypoalbuminemic alkalosis. Lactic acidosis was defined by BE_Lac_ < − 1.1 mmol/l (calculated BE_Lac_ for lactate at the upper limit of normal) and metabolic acidosis owing to unmeasured anions by BE_UMA_ < − 5 mmol/l.

### Statistical analysis

Data are presented as median and interquartile range (25–75% IQR), if not otherwise specified. PSM was used to minimize the confounding effect of severity of disease on acid–base status when comparing cirrhosis to non-cirrhosis patients. One-to-one PSM (1:1) was done by cirrhosis versus non-cirrhosis based on the following variables: SOFA score, need for mechanical ventilation and the presence of AKI. IBM SPSS 22 (with SPSS Python essentials and FUZZY extension command) was used for PSM. McNemar test was used for the comparison of binary and Wilcoxon’s signed-rank test for the comparison of metric variables between cirrhosis and matched controls. Nonparametric one-way ANOVA (Kruskal–Wallis test) with Dunn’s post hoc analysis was performed to assess differences in acid–base parameters between matched controls, cirrhosis patients without ACLF and ACLF patients. Within each group, comparisons were made using Chi-squared test or Mann–Whitney *U* test, as appropriate. Spearman’s rank correlation was used to assess correlations between metric variables. A receiver operating curve (ROC) analysis was performed, and the area under the ROC curve (AUROC) was calculated to evaluate the prognostic value of different metric variables. Impact of acid–base disorders on mortality was assessed using Cox regression. A *p* value < 0.05 is considered statistically significant. Statistical analysis was conducted using IBM SPSS Statistics version 22.

## Results

### Patients’ characteristics

One hundred and seventy-eight patients had liver cirrhosis, and 157 of these patients (88%) were admitted with ACLF. The remaining cirrhosis patients (*n* = 21, 12%) were admitted to ICU due to isolated non-kidney organ failure (*n* = 9), isolated cerebral failure (*n* = 4), bleedings (*n* = 4), infections (*n* = 3) and after surgery (*n* = 1); all of which did not fulfill criteria for ACLF. The control group consisted of 178 critically ill patients without acute or chronic liver disease. SAPS II score and SOFA score did not differ between patients with and without cirrhosis (Table 1).

**Table 1:** Baseline characteristics

Parameter	Propensity score-matched controls (*n* = 178)	Liver cirrhosis (*n* = 178)	*p* value
Age, years (IQR)	65 (55–75)	55 (48–62)	< 0.01
Male gender, *n* (%)	79 (44%)	82 (46%)	0.837
SOFA score (IQR)	12 (8–16)	13 (10–16)	0.084
SAPS II score (IQR)	59 (44–72)	62 (44–79	0.101
CLIF-SOFA score (IQR)	–	14 (11–16)	
ACLF grade			
No ACLF, *n* (%)		21 (12%)	
Grade I, *n* (%)	–	27 (15%)	
Grade II, *n* (%)	–	45 (25%)	
Grade III, *n* (%)	–	85 (48%)	
CLIF-C ACLF score (IQR)	–	56.5 (48.8–63.3)	
MELD score (IQR)	–	26 (20–35)	
Child–Pugh score (IQR)	–	11 (10–13)	
Acute kidney injury, *n* (%)	133 (75%)	138 (78%)	0.575
Vasopressor support, *n* (%)	154 (87%)	158 (89%)	0.596
Mechanical ventilation, *n* (%)	116 (65%)	101 (57%)	0.120
Laboratory parameters			
AST, U/l (IQR)	51 (30–116)	94 (54–204)	< 0.01
ALT, U/l (IQR)	32 (19–71)	43 (24–85)	0.096
Bilirubin, mg/dl (IQR)	1.0 (0.6–1.9)	5.4 (2.9–14.4)	< 0.01
INR (IQR)	1.2 (1.1–1.4)	1.8 (1.5–2.5)	< 0.01
Creatinine, mg/dl (IQR)	1.8 (1.2–2.7)	1.8 (1.1–3.1)	0.851
Outcome			
28-Day mortality, *n* (%)	54 (30%)	105 (59%)	< 0.01

*IQR* interquartile range, *SOFA* Sequential Organ Failure Assessment, *SAPS* Simplified Acute Physiology Score, *CLIF-SOFA* Chronic Liver Failure—Sequential Organ Failure Assessment, *ACLF* acute-on-chronic liver failure, *CLIF-C ACLF* CLIF consortium ACLF score, *MELD* Model of End-Stage Liver Disease, *AST* aspartate aminotransferase, *ALT* alanine aminotransferase, *INR* international normalized ratio

Causes of liver cirrhosis were alcoholic liver disease (*n* = 96, 54%), viral hepatitis (*n* = 31, 17%), combined alcoholic viral (*n* = 7, 4%), cryptogenic (*n* = 23, 13%), primary biliary cholangitis (*n* = 5, 3%) and others (*n* = 16, 9%). Triggers for occurrence ACLF were infections/sepsis (*n* = 110, 70%), bleeding (*n* = 23, 15%) and others.

Clinical and laboratory features of critically ill patients with and without cirrhosis are shown in Table 1.

### Acid–base disorders in critically ill patients with and without cirrhosis

Disturbances of acid–base balance were evident in the vast majority of our critically ill patients, irrespective of cirrhosis (Tables 2, 3). Critically ill patients (irrespective of cirrhosis) showed coexisting hyperchloremic acidosis and hypoalbuminemic alkalosis, mostly antagonizing each other in their contribution to total BE. In ACLF, we observed a marked metabolic acidosis owing to increased lactate levels, unmeasured anions and (to a lesser extent) dilutional acidosis. Both BE_UMA_ and SIG differed significantly between critically ill patients with ACLF and without liver disease, respectively, although the small difference in SIG may be clinically negligible (Table 2). In cirrhosis patients without ACLF, BE_UMA_ was significantly higher compared to patients with ACLF. The resulting metabolic acidosis in ACLF was partly compensated by coexisting respiratory alkalosis in its contribution to pH; however, increasing net metabolic acidosis is resulted in acidemia in patients with ACLF grade III (62%, Table 3). Metabolic differences between critically ill patients with and without cirrhosis tended to increase with the severity of disease, as indicated by SOFA score (Additional file 1: Figure S1).

**Table 2:** Acid–base parameters of critically ill patients with and without liver disease

Parameter	Propensity score-matched controls (*n* = 178)	Cirrhosis (*n* = 178)		Overall *p* value (Kruskal–Wallis)	Significant differences pairwise (Dunn’s post hoc)
		No ACLF (*n* = 21)	ACLF (*n* = 157)		
pH	7.36 (7.27 to 7.43)	7.44 (7.37 to 7.47)	7.35 (7.23 to 7.45)	< 0.01	No ACLF versus ACLF *p* < 0.01, matched controls versus no ACLF *p* < 0.01
PaCO_2_, mmHg	40.0 (33.1 to 49.0)	38.1 (30.0 to 44.2)	35.0 (28.5 to 44.6)	< 0.01	Matched controls versus ACLF *p* < 0.01
HCO_3_^−^	22.0 (19.0 to 25.3)	22.7 (20.3 to 24.0)	18.9 (14.7 to 24.0)	< 0.01	No ACLF versus ACLF *p* < 0.01, matched controls versus ACLF *p* < 0.01
BE	− 3.5 (− 7.4 to 0.8)	− 1.2 (− 3.9 to 1.7)	− 7.0 (− 12.6 to − 0.5)	< 0.01	No ACLF versus ACLF *p* < 0.01, matched controls versus ACLF *p* < 0.01
BE_Na_	− 0.3 (− 1.5 to 0.9)	− 0.9 (− 1.8 to 0.3)	− 1.2 (− 3.0 to 0.3)	< 0.01	Matched controls versus ACLF *p* < 0.01
BE_Cl_	− 5.7 (− 8.3 to − 2.7)	− 5.2 (− 8.5 to − 1.4)	− 4.5 (− 7.3 to 0.7)	0.062	
BE_Alb_	4.2 (2.9 to 5.3)	5.2 (3.9 to 6.3)	4.9 (3.8 to 6.2)	< 0.01	Matched controls versus ACLF *p* < 0.01
BE_lactate_	− 0.6 (− 1.9 to − 0.2)	− 0.9 (− 1.9 to − 0.4)	− 2.7 (− 6.0 to − 0.9)	< 0.01	No ACLF versus ACLF *p* < 0.01, matched controls versus ACLF *p* < 0.01
BE_UMA_	− 0.3 (− 3.7 to 2.7)	1.5 (− 0.7 to 4.3)	− 1.8 (− 6.1 to 1.9)	< 0.01	No ACLF versus ACLF *p* < 0.01, matched controls versus ACLF *p* < 0.01
SIDe, mEq/l	33 (30 to 37)	32 (30 to 37)	29 (25 to 34)	< 0.01	No ACLF versus ACLF *p* < 0.05, matched controls versus ACLF *p* < 0.01
SIDa, mEq/l	41 (37 to 43)	40 (36 to 44)	39 (35 to 42)	< 0.01	Matched controls versus ACLF *p* < 0.01
SIG, mEq/l	7 (4 to 10)	7 (5 to 8)	8 (6 to 11)	< 0.01	No ACLF versus ACLF *p* < 0.05, matched controls versus ACLF *p* < 0.01
Na	138 (134 to 142)	136 (133 to 140)	135 (129 to 140)	< 0.01	Matched controls versus ACLF *p* < 0.01
Cl	106 (102 to 109)	105 (99 to 108)	102 (96 to 108)	< 0.01	Matched controls versus ACLF *p* < 0.01
Cl_Na corrected_	107 (104 to 109)	106 (102 to 110)	106 (100 to 108)	0.075	
Ca total	2.1 (2.0 to 2.2)	2.0 (1.9 to 2.1)	2.0 (1.9 to 2.2)	0.191	
Ca ionized	1.1 (1.1 to 1.2)	1.2 (1.1 to 1.2)	1.1 (1.0 to 1.2)	< 0.01	No ACLF versus ACLF *p* < 0.05, matched controls versus ACLF *p* < 0.01
Mg	0.9 (0.7 to 1.0)	0.7 (0.7 to 0.9)	0.9 (0.7 to 1.0)	< 0.05	No ACLF versus ACLF *p* < 0.05
Albumin, g/l	28.5 (24.3 to 33.8)	25.8 (21.8 to 30.5)	25.6 (21.1 to 30.3)	< 0.01	Matched controls versus ACLF *p* < 0.01
Lactate	1.4 (1.0 to 2.7)	1.7 (1.2 to 2.7)	3.5 (1.7 to 6.8)	< 0.01	No ACLF versus ACLF *p* < 0.01, matched controls versus ACLF *p* < 0.01

*ACLF* acute-on-chronic liver failure, *PaCO*_*2*_ partial pressure of arterial carbon dioxide, *HCO*_*3*_^*−*^ bicarbonate, *BE* base excess, *SBE* standard base excess, *BE*_*Na*_ BE caused by free water effect, *BE*_*Cl*_ BE caused by changes in chloride, *BE*_*Alb*_ BE caused by albumin effect, *BE*_*lactate*_ BE attributable to lactate elevation, *BE*_*UMA*_ BE attributable to unmeasured anions, *SIDe* effective strong ion difference, *SIDa* apparent strong ion difference, *SIG* strong ion gap, *Na* sodium, *Cl* chloride, *Ca* calcium, all values are given in mmol/l with interquartile range (IQR), unless otherwise indicated

**Table 3:** Acid–base disorders stratified according to the presence of cirrhosis and ACLF

Metabolic disturbances on admission	Propensity score-matched controls (*n* = 178)	Cirrhosis (*n* = 178)				*p* value for overall cirrhosis versus matched controls	*p* value for the effect of ACLF category*
		Overall cirrhosis (*n* = 178)	ACLF category				
			No ACLF (*n* = 21)	ACLF grade 1 and 2 (*n* = 72)	ACLF grade III (*n* = 85)		
Acidemia	87 (49%)	86 (48%)	4 (19%)	29 (40%)	53 (62%)	1.00	< 0.01
Alkalemia	35 (20%)	52 (29%)	11 (52%)	21 (29%)	20 (24%)	< 0.05	< 0.05
Respiratory acidosis	64 (36%)	41 (23%)	3 (14%)	13 (18%)	25 (29%)	< 0.05	0.052
Respiratory alkalosis	55 (31%)	88 (49%)	10 (48%)	40 (56%)	38 (45%)	< 0.01	0.338
Metabolic acidosis	89 (50%)	112 (63%)	7 (33%)	45 (63%)	60 (71%)	< 0.05	< 0.01
Metabolic alkalosis	38 (21%)	33 (19%)	4 (19%)	16 (22%)	13 (15%)	0.596	0.365
Dilutional acidosis	1 (0.6%)	11 (6%)	0	4 (6%)	7 (8%)	< 0.01	0.205
Concentrational alkalosis	2 (1.1%)	6 (3%)	0	6 (8%)	0	0.289	0.136
Hyperchloremic acidosis	98 (55%)	78 (44%)	11 (52%)	32 (44%)	35 (41%)	< 0.05	0.399
Hypochloremic alkalosis	7 (4%)	15 (8%)	0	8 (11%)	7 (8%)	0.134	0.702
Hypoalbuminemic alkalosis	58 (33%)	86 (48%)	11 (52%)	36 (50%)	39 (46%)	< 0.01	0.516
Acidosis owing to unmeasured anions	32 (18%)	48 (27%)	1 (5%)	16 (22%)	31 (37%)	0.061	< 0.01
Lactic acidosis	65 (37%)	118 (66%)	7 (33%)	44 (61%)	67 (79%)	< 0.01	< 0.01

All values are given in number (*n*) and percent (%)

*ACLF* acute-on-chronic liver failure

*p value calculated by univariate ordinal logistic regression

Both SIG and BE_UMA_ were associated with renal impairment (Additional file 2: Figure S2). Overall (*n* = 356), SIG was significantly higher and BE_UMA_ significantly lower in patients presenting with AKI as compared to those without [8.4 (IQR 6.0–11.1) mmol/l vs. 5.4 (IQR 2.7–7.5) mmol/l and − 2.0 (IQR − 6.0 to 1.4) mmol/l vs. 2.8 (IQR − 0.3 to 5.6) mmol/l; *p* < 0.01 for both].

Lactate levels were significantly elevated in critically ill patients with liver cirrhosis compared to those without [3.0 (IQR 1.7–6.1) mmol/l vs. 1.4 (IQR 1.0–2.7) mmol/l; *p* < 0.01]. Additionally, lactate levels were higher in patients receiving vasopressors compared to those without [2.3 (IQR 1.3–4.6) mmol/l vs. 1.2 (IQR 0.9–1.8) mmol/l; *p* < 0.01]. Lactate levels increased with SOFA score in cirrhotic and non-cirrhotic patients (Additional file 1: Figure S1). Accordingly, highest lactate levels were observed in patients with ACLF (Table 2). Lactate levels correlated with bilirubin (*r* = 0.41) and international normalized ratio (INR, *r* = 0.46), respectively, but also weakly with serum creatinine (*r* = 0.17); *p* < 0.01 for all.

**Fig. 1:**
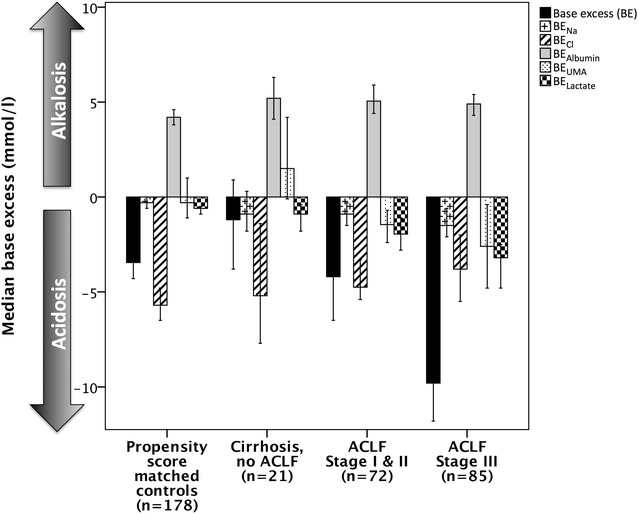
Disequilibrium in acid–base status in critically ill patients with liver cirrhosis, acute-on-chronic liver failure (ACLF) and without chronic liver disease. Results displayed as median and 95% CI; associations of base excess and its subcomponents with ACLF stage in cirrhosis patients assessed by univariate ordinal regression: BE *p* < 0.001, BE_Na_ *p* = 0.074, BE_Cl_ *p* = 0.728, BE_Alb_ *p* = 0.295, BE_Lac_ *p* < 0.001, BE_UMA_ *p* < 0.05. Differences between cirrhosis and control patients are illustrated in Table 2

Metabolic acid–base characteristics of critically ill patients with and without liver disease are illustrated in Fig. 1 and Additional file 1: Figure S1.

### Acid–base equilibrium and outcome in patients with liver cirrhosis

**Fig. 2:**
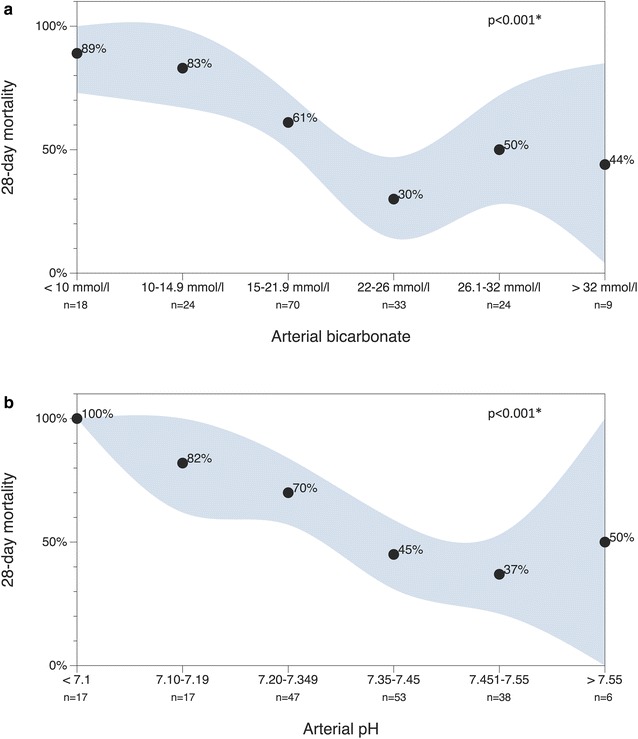
Association of bicarbonate (**a**) and pH (**b**) with 28-day mortality in critically ill patients with liver cirrhosis. Black dots: observed 28-day mortality rate; gray area: 95% confidence interval. *p values calculated by Chi-square test

In particular, metabolic acidosis and acidemia, respectively, were linked to 28-day mortality in cirrhosis (Fig. 2, Additional file 3: Table S1). Accordingly, arterial pH values < 7.1 on admission were associated with 100% and HCO_3_^−^ values < 10 mmol/l with 89% 28-day mortality, respectively (Fig. 2).

Similarly, BE showed a strong association with 28-day mortality (Additional file 3: Table S1). Analysis of the BE subgroups revealed that the impact on mortality in cirrhosis was primarily caused by lactate and unmeasured anions (Table 4). This effect remained significant after correction for demographics, ACLF grade and the presence of infection/sepsis (Table 4). AUROCs for admission lactate/BE_Lac_ and BE_UMA_ in prediction of 28-day mortality in critical ill patients with liver cirrhosis were 0.744 (95% CI 0.671–0.816) and 0.692 (95% CI 0.613–0.770), respectively (*p* < 0.001 for both). Thus, the predictive potential of admission arterial lactate levels regarding 28-day mortality in critically ill cirrhosis patients at the ICU was comparable to SOFA score [AUROC 0.780 (95% CI 0.713–0.847)].

**Table 4:** Cox regression model for risk factors for mortality in critically ill patients with liver cirrhosis

Parameter	Hazard ratio (95% CI)	
	Univariate	Multivariate
Age	1.02 (1.00–1.04)*	1.02 (1.00–1.04)*
Sex (male gender)	0.75 (0.51–1.11)	0.77 (0.51–1.15)
Liver disease		
ACLF grade 1 versus no ACLF	1.80 (0.63–5.19)	1.36 (0.47–4.01)
ACLF grade 2 versus no ACLF	2.02 (0.76–5.37)	1.44 (0.53–3.94)
ACLF grade 3 versus no ACLF	5.52 (2.22–13.74)**	3.68 (1.42–9.52)**
Sepsis/infection	1.69 (1.09–2.61)*	1.21 (0.76–1.92)
Base excess		
BE_Na_	0.96 (0.90–1.03)	0.96 (0.89–1.04)
BE_Cl_	1.00 (0.98–1.03)	0.97 (0.93–1.00)
BE_Alb_	0.91 (0.81–1.02)	0.89 (0.79–1.00)
BE_UMA_	0.95 (0.93–0.97)**	0.96 (0.92–0.99)*
BE_lactate_	0.88 (0.85–0.92)**	0.92 (0.88–0.97)**

*ACLF* acute-on-chronic liver failure, *BE*_*Na*_ BE caused by free water effect, *BE*_*Cl*_ BE caused by changes in chloride, *BE*_*Alb*_ BE caused by albumin effect, *BE*_*lactate*_ BE attributable to lactate elevation, *BE*_*UMA*_ BE attributable to unmeasured anions

*p value < 0.05; **p value < 0.01

In our matched controls, we observed no significant effect of acidemia, alkalemia, lactic acidosis and net metabolic acidosis, respectively, on 28-day mortality. Yet, pH values differed significantly between non-cirrhosis 28-day survivors and non-survivors [7.37 (IQR 7.29–7.44) vs. 7.34 (IQR 7.22–7.34), *p* < 0.05, Additional file 3: Table S1]. Acidosis attributable to unmeasured anions was associated with 28-day mortality in our propensity score-matched controls; however, BE_UMA_ did not differ significantly between non-cirrhotic 28-day survivors and non-survivors (Additional file 3: Table S1). Moreover, admission arterial lactate levels differed significantly between non-cirrhosis 28-day survivors and non-survivors [1.4 (IQR 0.9–2.4) mmol/l vs. 1.7 (IQR 1–4.1) mmol/l; *p* < 0.05]. Yet, the association between metabolic derangement and outcome was more distinct in cirrhosis patients (Additional file 3: Table S1).

## Discussion

Disturbances in acid–base equilibrium are common in critical illness [16]. In this study, we demonstrate that critically ill patients with cirrhosis and ACLF, respectively, differentiate considerably from patients without hepatic impairment in terms of acid–base balance.

In accordance with earlier reports, we observed in our cohort a marked hyperchloremic acidosis with coexisting hypoalbuminemic alkalosis [8, 9, 11]. This phenomenon, however, was not limited to patients with cirrhosis and should therefore not be considered an exclusive acid–base pattern of liver disease. Instead, this seems to be a characteristic pattern of critical illness per se [3]. Yet, hypoalbuminemia and resulting alkalosis were most pronounced in patients with ACLF. However, the main distinguishing metabolic acid–base characteristic between critically ill patients with and without cirrhosis was a marked metabolic acidosis attributable to an increased lactate (and unmeasured anions). In cirrhosis, coexisting respiratory alkalosis partly compensated for metabolic acidosis, thereby resulting in almost normal pH values. However, respiratory alkalosis failed to compensate for net metabolic acidosis in patients with ACLF.

Increased lactate levels in critically ill patients can result from both increased production (e.g., tissue malperfusion, impaired cellular oxygen metabolism during sepsis, hypermetabolic states) and reduced lactate clearance (e.g., loss of functioning hepatocytes in acute hepatic injury or chronic liver disease) [32–34]. The liver not only is a crucial player in the disposal of lactate, but may also become a net producer of lactate, especially during hepatic parenchymal hypoxia. Although lactic acidosis has been described in the literature in critical ill patients with cirrhosis [7, 8], this is the first study investigating the association of metabolic disturbances with ACLF compared to a matched cohort of critically ill patients without liver disease. Indeed, the extent of lactic acidosis was directly associated with ACLF grade. Accordingly, lactic acidosis was present in almost 80% of all patients with ACLF grade III. Moreover, lactate levels were correlated with INR and bilirubin, thereby suggesting that lactate levels are directly related to liver function. Vasopressor support and severity of disease (as reflected by SOFA score) were also significantly associated with increased lactate levels. In sum, our data suggest that a combination of hepatic impairment and tissue hypoxia may contribute to lactic acidosis in critically ill patients with liver cirrhosis.

Great effort has been put in revealing the nature of unmeasured anions in critical illness [2, 35–38]. Still, source and clinical implications of unmeasured anions are incompletely understood [39, 40]. Recently, it was shown in a large cohort of critically ill patients that increased concentrations of unmeasured anions were independently associated with increased mortality [41]. Citrate, acetate, fumarate, *α*-ketoglutarate and urate have been identified as potential candidates contributing to acidosis associated with high SIG in hemorrhagic shock [36]. Apart from states of shock, renal failure has been linked to increased levels of unmeasured anions in several studies [8, 42, 43]. As compared to non-ACLF cirrhosis patients, the presence of ACLF was associated with an increase in unmeasured anions, as reflected by BE_UMA_ and SIG. Both variables were strongly associated with acute kidney injury. Patients with liver cirrhosis are especially susceptible to renal failure [44–47], and renal impairment constitutes a central criterion for ACLF [20]. In sum, our findings indicate that impairment of renal function, rather than “hepatic failure,” may be responsible for the increase in levels of unmeasured anions observed in patients with ACLF.

In the present study, metabolic acidosis and acidemia, respectively, were associated with increased 28-day mortality in liver cirrhosis. Accordingly, 28-day mortality rate was 91% in cirrhosis patients with arterial pH values < 7.2 and 86% in those with arterial HCO_3_^−^ values < 15 mmol/l. Lactic acidosis and acidosis attributable to unmeasured anions were identified as main contributors to acid–base imbalance in critically ill patients with liver cirrhosis. Earlier studies have challenged the prognostic value of unmeasured anions or lactate in critically ill patients [40]. Yet, the relationship between lactate levels, unmeasured anions and mortality and poor outcome has been described multiply in the literature [7, 8, 32, 33, 48], and lactate levels have recently been suggested as a parameter, indicating severity of disease in patients with chronic liver disease [49]. In our critically ill cirrhosis patients, we observed a dramatic independent impact of both lactate and BE_UMA_ on 28-day mortality. Thus, acid–base status in critically ill patients with cirrhosis and ACLF, respectively, is an early and independent predictor of outcome (Fig. 2). By contrast, acid–base status was of poor prognostic value in our propensity score-matched controls. This may be attributable to the fact that our control patients were matched to critically ill cirrhosis patients, thereby resulting in the exclusion of less severely ill non-cirrhosis patients with better acid–base profiles and lower mortality rates.

This study has strengths and limitations. First, this is a post hoc analysis; however, our study comprises structured acid–base analyses from a large cohort of critically ill patients stratified according to the presence of liver cirrhosis. Second, this study was performed in patients admitted to the ICU. Thus, our findings may not entirely reflect acid–base status of cirrhotic patients treated at normal wards. However, our study also incorporates cirrhosis patients without ACLF and patients of all ACLF categories. Third, there are pros and cons of propensity score matching. In this study, we have decided to use propensity score-matched controls in order to minimize the confounding effect of severity of disease on acid–base balance. Although we were able to achieve good comparability, inherent differences between cirrhotic and non-cirrhotic patients affecting acid base balance cannot be entirely abolished by matching procedures. Moreover, the loss of heterogeneity (by selection of the most severely ill patients) hampers survival analyses in the control group. Fourth, residual confounding is, as always, a matter of concern and cannot be entirely excluded. Future studies should confirm these results and focus on therapeutic implications for patients with liver disease at the ICU.

## Conclusions

In conclusion, we could demonstrate that hyperchloremic acidosis and hypoalbuminemic alkalosis coexist in critically ill patients, including those with liver cirrhosis. In cirrhosis, but particularly in ACLF, net metabolic acidosis was caused by lactate and unmeasured anions. Lactate was linked to liver function and vasopressor use, whereas unmeasured anions were strongly related to acute kidney injury. Metabolic differences between cirrhosis and non-cirrhosis critically ill patients increase with the severity of disease, resulting in pronounced acidemia in cirrhosis patients with ACLF. Acidemia and metabolic acidosis, respectively, were associated with poor outcome in cirrhosis patients. Lactate and BE_UMA_ were identified as independent predictors of 28-day mortality in critically ill patients with liver cirrhosis and ACLF.

### Additional files

**Additional file 1: Figure S1.**Acid–base disturbances and their relation to severity of disease in critically ill patients with and without liver cirrhosis. Overall following parameter differed significantly between cirrhosis and non-cirrhosis patients (Wilcoxon’s signed-rank test): BE (*p* < 0.01), lactate (*p* < 0.001), BE_UMA_ (*p* < 0.05), SIG (*p* < 0.01) and PaCO_2_ (*p* < 0.01), but not pH (*p* = 0.624). *p values between regression slopes were obtained from linear regression models with interaction terms

**Additional file 2: Figure S2.**Base excess attributable to unmeasured anions (BE_UMA_) and strong ion gap (SIG) are associated with acute kidney injury in critically ill patients with and without cirrhosis. BE_UMA_ (*p* < 0.05) and SIG (*p* < 0.01) differed significantly between patients with and without cirrhosis, but correlated significantly with stage of acute kidney injury in both groups (*p* < 0.001). The association of BE_UMA_ and SIG, respectively, with acute kidney injury did not differ between patients with and without cirrhosis (*p* = 0.994 and 0.824)

**Additional file 3: Table S1.**

## Appendix

BE subcomponents reflecting the contributions of sodium (BE_Na_), chloride (BE_Cl_), albumin (BE_Na_), lactate (BE_Lac_) and unmeasured anions (BE_UMA_), according to Gilfix et al. [31]:

(A) Na^+^ was used to assess BE caused by free water effect (dilution)
  $${\text{BE}}_{\text{Na}} = 0.3 \times \left( {{\text{Na}}^{ + } - {\text{Na}}_{\text{normal}}^{ + } } \right)\quad \left( {{\text{Na}}^{ + }_{\text{normal}} = 139\;{\text{mmol/l}}} \right)$$
(B) After correction Cl^−^ for changes in free water (Cl_Na corrected_^−^)
  $${\text{Cl}}^{ - }_{{{\text{Na}}\;{\text{corrected}}}} = {\text{Cl}}^{ - } \times \frac{{{\text{Na}}^{ + }_{\text{normal}} }}{{{\text{Na}}^{ + } }}$$
  BE attributable to chloride (BE_Cl_) was calculated:
  $${\text{BE}}_{\text{Cl}} = {\text{Cl}}^{ - }_{\text{normal}} - {\text{Cl}}^{ - }_{{{\text{Na}}\;{\text{corrected}}}} \quad \left( {{\text{Cl}}^{ - }_{\text{normal}} = 101\;{\text{mmol/l}}} \right)$$
(C) BE attributable to albumin was calculated as follows [28]:
  $${\text{BE}}_{\text{Alb}} = \left( {0.148 \times {\text{pH}} - 0.818} \right) \times \left( {{\text{Alb}}_{\text{normal}} - {\text{Alb}}_{\text{observed}} } \right)\quad \left( {{\text{Alb}}_{\text{normal}} = 44.4\;{\text{g/l}}} \right)$$
(D) BE due to lactate was calculated:
  $${\text{BE}}_{\text{Lac}} = {\text{lactate}}_{\text{normal}} - {\text{lactate}}_{\text{observed}} \quad \left( {{\text{lactate}}_{\text{normal}} = 0.8\;{\text{mmol/l}}} \right)$$
(E) Changes in BE not related to the aforementioned factors correspond to UMA, which are quantified as follows:
  $${\text{BE}}_{\text{UMA}} = {\text{BE}} - \left( {{\text{BE}}_{\text{Na}} + {\text{BE}}_{\text{Cl}} + {\text{BE}}_{\text{Alb}} + {\text{BE}}_{\text{Lac}} } \right)$$

## Abbreviations

ICU: intensive care unit
ALF: acute liver failure
ACLF: acute-on-chronic liver failure
PSM: propensity score matching
SAPS II: Simplified Acute Physiology Score II
SOFA: Sequential Organ Failure Assessment
AKI: acute kidney injury
KDIGO: Kidney Disease: Improving Global Outcomes
CLIF: chronic liver failure
EASL: European Association for the Study of the Liver
CLIF-SOFA: Chronic Liver Failure Sequential Organ Failure Assessment
CLIF-C ACLF: chronic liver failure consortium acute-on-chronic liver failure score
PaCO_2_: partial pressure of arterial carbon dioxide
Ca^2+^: total calcium
K^+^: potassium
Cl^−^: chloride
Mg^2+^: magnesium
Pi: inorganic phosphate
Alb: albumin
BUN: blood urea nitrogen
AST: aspartate aminotransferase
ALT: alanine aminotransferase
HCO3^−^: bicarbonate
BE: base excess
SID_a_: apparent strong ion difference
SID_e_: effective strong ion difference
SIG: strong ion gap
UMA: unmeasured anions
BE_Na_: base excess attributable to sodium
BE_Cl_: base excess attributable to chloride
BE_Alb_: base excess attributable to albumin
BE_Lac_: base excess attributable to lactate
BE_UMA_: base excess attributable to unmeasured anions
IQR: interquartile range
ROC: receiver operating characteristic
AUROC: area under the receiver operating characteristic curve
