# Supplementary figures and images for: Acid–base status and its clinical implications in critically ill patients with cirrhosis, acute-on-chronic liver failure and without liver disease
Source: Ann Intensive Care. 2018 Apr 19;8:48. doi: 10.1186/s13613-018-0391-9 (PMC5908779; doi:10.1186/s13613-018-0391-9)

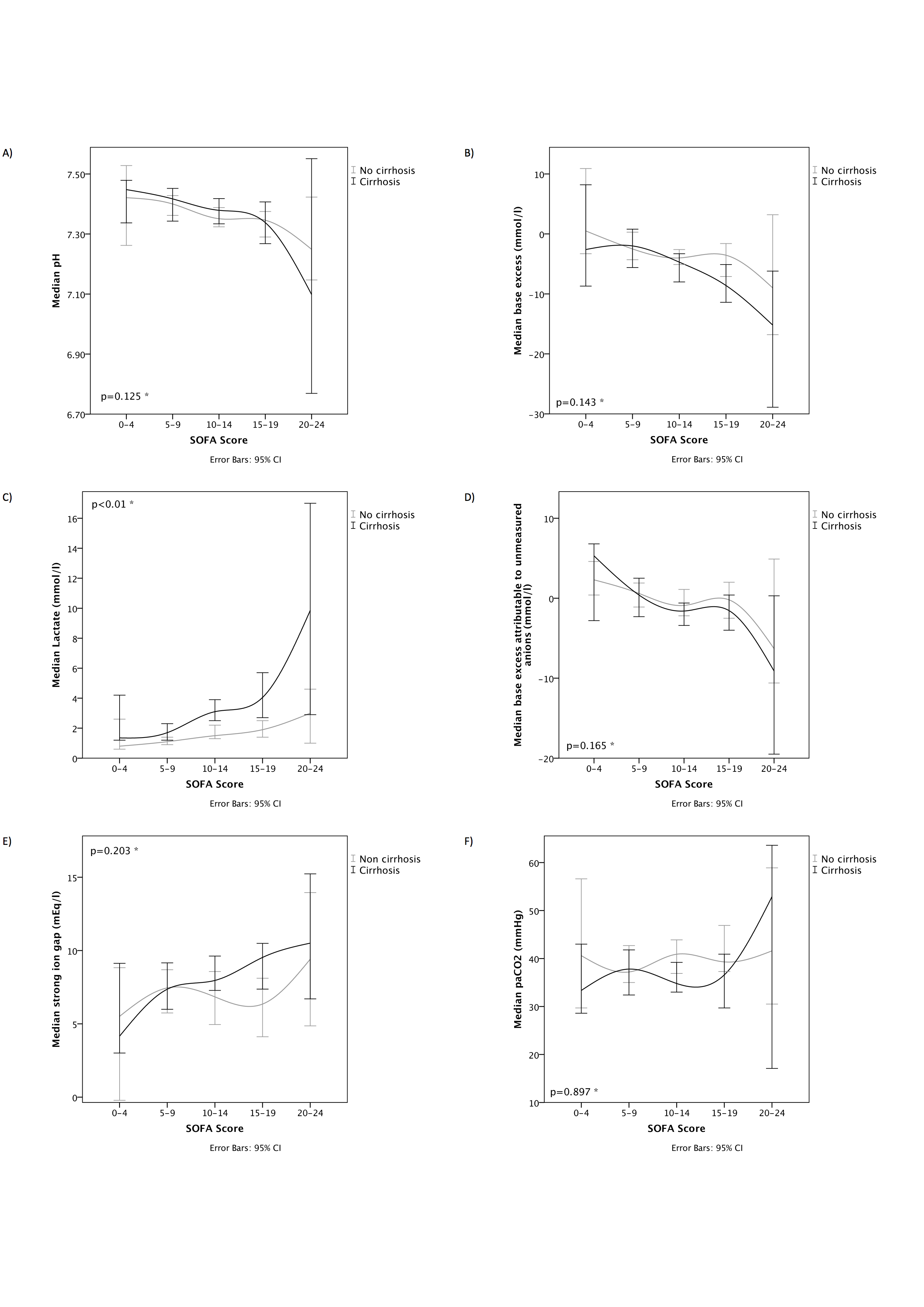

Supplement: Supplementary file 1 — Additional file 1: Figure S1.Acid–base disturbances and their relation to severity of disease in critically ill patients with and without liver cirrhosis. Overall following parameter differed significantly between cirrhosis and non-cirrhosis patients (Wilcoxon’s signed-rank test): BE (p < 0.01), lactate (p < 0.001), BEUMA (p < 0.05), SIG (p < 0.01) and PaCO2 (p < 0.01), but not pH (p = 0.624). *p values between regression slopes were obtained from linear regression models with interaction terms [file 13613_2018_391_MOESM1_ESM.tiff]

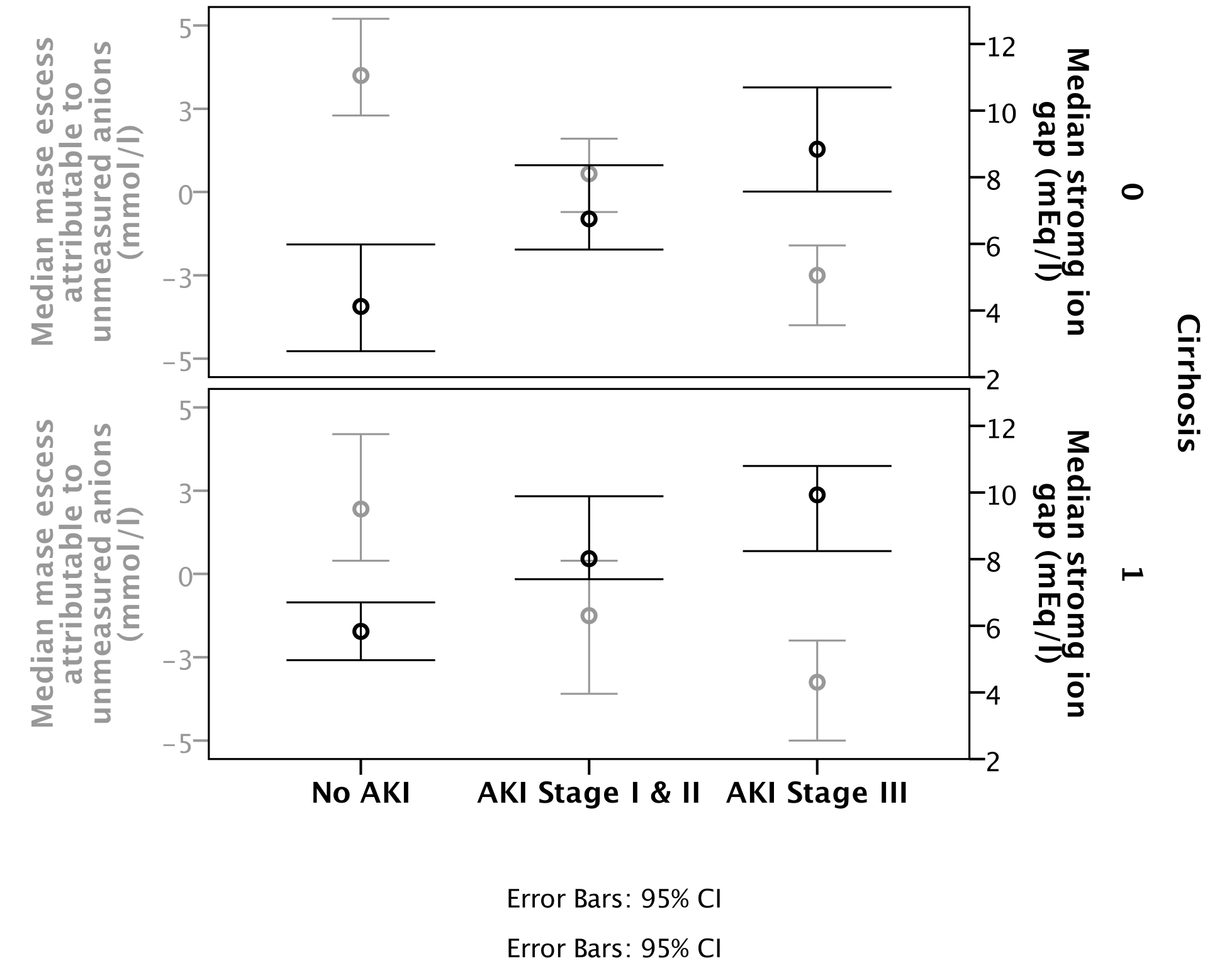

Supplement: Supplementary file 2 — Additional file 2: Figure S2.Base excess attributable to unmeasured anions (BEUMA) and strong ion gap (SIG) are associated with acute kidney injury in critically ill patients with and without cirrhosis. BEUMA (p < 0.05) and SIG (p < 0.01) differed significantly between patients with and without cirrhosis, but correlated significantly with stage of acute kidney injury in both groups (p < 0.001). The association of BEUMA and SIG, respectively, with acute kidney injury did not differ between patients with and without cirrhosis (p = 0.994 and 0.824) [file 13613_2018_391_MOESM2_ESM.tiff]
