# Supplementary material for: Acid–base status and its clinical implications in critically ill patients with cirrhosis, acute-on-chronic liver failure and without liver disease
Source: Ann Intensive Care. 2018 Apr 19;8:48. doi: 10.1186/s13613-018-0391-9 (PMC5908779; doi:10.1186/s13613-018-0391-9)
Supplement: Supplementary file 3 — Additional file 3: Table S1. [file 13613_2018_391_MOESM3_ESM.docx]

**Supplemental Table 1** Acid-base disturbances in ICU patients with and without cirrhosis stratified according to 28-day mortality

| **Acid-base disturbances on admission** | **Propensity score matched controls (n=178)** | | **p-value** |  | **Cirrhosis (n=178)** | | **p-value** |
| --- | --- | --- | --- | --- | --- | --- | --- |
|  | **28-day survivors**  **(n=124)** | **28-day non-survivors**  **(n=54)** |  |  | **28-day survivors**  **(n=73)** | **28-day non-survivors**  **(n=105)** |  |
| Number of patients*, n (%)* | 124 (70%) | 54 (30%) |  |  | 73 (41%) | 105 (59%) |  |
| Acidaemia*, n (%)* | 57 (46%) | 30 (56%) | 0.239 |  | 20 (27%) | 66 (63%) | <0.01 |
| Alkalaemia*, n (%)* | 28 (23%) | 7 (13%) | 0.138 |  | 30 (41%) | 22 (21%) | <0.01 |
| Respiratory acidosis*, n (%)* | 45 (36%) | 19 (35%) | 0.888 |  | 14 (19%) | 27 (26%) | 0.308 |
| Respiratory alkalosis*, n (%)* | 40 (32%) | 15 (28%) | 0.552 |  | 37 (51%) | 51 (49%) | 0.781 |
| Metabolic acidosis*, n (%)* | 60 (48%) | 29 (54%) | 0.514 |  | 33 (45%) | 79 (75%) | <0.01 |
| Metabolic alkalosis*, n (%)* | 29 (23%) | 9 (17%) | 0.314 |  | 17 (23%) | 16 (15%) | 0.174 |
| Dilutional acidosis*, n (%)* | 1 (1%) | 0 | 1 |  | 4 (6%) | 7 (7%) | 1 |
| Concentrational alkalosis*, n (%)* | 2 (2%) | 0 | 1 |  | 3 (4%) | 3 (3%) | 0.690 |
| Hyperchloremic acidosis*, n (%)* | 70 (57%) | 28 (52%) | 0.571 |  | 36 (49%) | 42 (40%) | 0.218 |
| Hypochloremic alkalosis*, n (%)* | 6 (5%) | 1 (2%) | 0.677 |  | 6 (8%) | 9 (9%) | 1 |
| Hypoalbumonemic alkalosis*, n (%)* | 40 (32%) | 18 (33%) | 0.888 |  | 39 (53%) | 47 (45%) | 0.255 |
| Acidosis owing to unmeasured anions*, n (%)* | 15 (12%) | 17 (32%) | <0.01 |  | 10 (14%) | 38 (36%) | <0.01 |
| Lactic acidosis*, n (%)* | 41 (33%) | 24 (44%) | 0.147 |  | 33 (45%) | 85 (81%) | <0.01 |
|  |  |  |  |  |  |  |  |
| **Metabolic acid-base parameters** |  |  |  |  |  |  |  |
| pH, *median (IQR)* | 7.37 (7.29-7.44) | 7.34 (7.22-7.41) | <0.05 |  | 7.42 (7.35-7.48) | 7.30 (7.17 to 7.43) | <0.01 |
| HCO_3_^-^ (mmol/l), *median (IQR)* | 22.2 (19.4-25.4) | 21.6 (17.5-24.1) | 0.144 |  | 22.4 (18.3-25.8) | 17.3 (12.6-22.0) | <0.01 |
| BE (mmol/l), *median (IQR)* | -3.1 (-7.1 to 1.5) | -4.3 (-9.1 to 0.53) | 0.061 |  | -1.9 (-7.0 to 2.4) | -8.9 (-15.3 to -3.6) | <0.01 |
| BE_Na_ (mmol/l), *median (IQR)* | -0.3 (-1.4 to 0.9) | -0.2 (-1.8 to 0.7) | 0.610 |  | -0.9 (-2.0 to 0.3) | -1.2 (-3.3 to 0.6) | 0.309 |
| BE_Cl_ (mmol/l), *median (IQR)* | -6.0 (-8.6 to -2.7) | -5.6 (-7.9 to -2.7) | 0.644 |  | -5.0 (-5.5 to -1.0) | -3.5 (-7.1 to 0.9) | 0.115 |
| BE_Alb_ (mmol/l), *median (IQR)* | 4.1 (2.9-5.4) | 4.5 (2.7 to 5.3) | 0.893 |  | 5.3 (4.0 to 6.6) | 4.9 (3.8 to 6.0) | 0.090 |
| BE_UMA_ (mmol/l), *median (IQR)* | 0.4 (-2.7 to 2.8) | -1.7 (-6.2 to 2.4) | 0.088 |  | 1.0 (-2.6 to 3.6) | -2.6 (-8.4 to 0.4) | <0.01 |
| BE_lactate_ (mmol/l), *median (IQR)* | -0.6 (-1.6 to -0.1) | -0.9 (-3.3 to -0.2) | <0.05 |  | -1.0 (-3.1 to -0.4) | -3.5 (-8.0 to -1.7) | <0.01 |
| IQR, interquartile range; HCO_3_^-^, bicarbonate; BE_Na_, BE caused by free water effect; BE_Cl_, BE caused by changes in chloride; BE_Alb_, BE caused by albumin effect; BE_UMA_, BE attributable to unmeasured anions; BE_lactate_, BE attributable to lactate-elevation. | | | | | | | |
